# Nested Session Types

**DOI:** 10.1007/978-3-030-72019-3_7

**Published:** 2021-03-23

**Authors:** Ankush Das, Henry DeYoung, Andreia Mordido, Frank Pfenning

**Affiliations:** Imperial College, London, UK; 9 Carnegie Mellon University, Pittsburgh, PA USA; 10 LASIGE, Faculdade de Ciências, Universidade de Lisboa, Lisbon, Portugal

## Abstract

Session types statically describe communication protocols between concurrent message-passing processes. Unfortunately, parametric polymorphism even in its restricted prenex form is not fully understood in the context of session types. In this paper, we present the metatheory of session types extended with prenex polymorphism and, as a result, nested recursive datatypes. Remarkably, we prove that type equality is decidable by exhibiting a reduction to trace equivalence of deterministic first-order grammars. Recognizing the high theoretical complexity of the latter, we also propose a novel type equality algorithm and prove its soundness. We observe that the algorithm is surprisingly efficient and, despite its incompleteness, sufficient for all our examples. We have implemented our ideas by extending the Rast programming language with nested session types. We conclude with several examples illustrating the expressivity of our enhanced type system.

### 1 Introduction

Session types express and enforce interaction protocols in message-passing systems [29,44]. In this work, we focus on *binary session types* that describe bilateral protocols between two endpoint processes performing dual actions. Binary session types obtained a firm logical foundation since they were shown to be in a Curry-Howard correspondence with linear logic propositions [7,8,47]. This allows us to rely on properties of cut reduction to derive type safety properties such as *progress (deadlock freedom)* and *preservation (session fidelity)*, which continue to hold even when extended to recursive types and processes [17].

However, the theory of session types is still missing a crucial piece: a general understanding of prenex (or ML-style) parametric polymorphism, encompassing recursively defined types, polymorphic type constructors, and nested types. We abbreviate the sum of these features simply as *nested types* [3]. Prior work has restricted itself to parametric polymorphism either: in prenex form without nested types [26,45]; with explicit higher-rank quantifiers [6,38] (including bounded ones [24]) but without general recursion; or in specialized form for iteration at the type level [46]. None of these allow a free, *nested* use of polymorphic type constructors combined with prenex polymorphism.

In this paper, we develop the metatheory of this rich language of nested session types. Nested types are reasonably well understood in the context of functional languages [3,32] and have a number of interesting applications [10,28,37]. One difficult point is the interaction of nested types with polymorphic recursion and type inference [36]. By adopting bidirectional type-checking we avoid this particular set of problems altogether, at the cost of some additional verbosity. However, we have a new problem namely how to handle type equality ($\equiv$) given that session type definitions are generally *equirecursive* and *not generative*. This means that even before we consider nesting, with the definitions

$$\mathsf{list}[\alpha] = \oplus\{\mathbf{nil} : \mathbf{1}, \mathbf{cons} : \alpha \otimes \mathsf{list}[\alpha]\} \qquad \mathsf{list}'[\alpha] = \oplus\{\mathbf{nil} : \mathbf{1}, \mathbf{cons} : \alpha \otimes \mathsf{list}'[\alpha]\}$$

we have $\mathsf{list}[A] \equiv \mathsf{list}'[B]$ and also $\mathsf{list}[\mathsf{list}'[A]] \equiv \mathsf{list}'[\mathsf{list}[B]]$ provided $A \equiv B$. The reason is that both types specify the same communication behavior—only their name (which is irrelevant) is different. As the second of these equalities shows, deciding the equality of nested occurrences of type constructors is inescapable: allowing type constructors (which are necessary in many practical examples) means we also have to solve type equality for nested types. For example, the types $\mathsf{Tree}[\alpha]$ and $\mathsf{STree}[\alpha][\kappa]$ represent binary trees and their faithfully (and efficiently) serialized form respectively.

$$\mathsf{Tree}[\alpha] = \oplus\{\mathbf{node} : \mathsf{Tree}[\alpha] \otimes \alpha \otimes \mathsf{Tree}[\alpha], \mathbf{leaf} : \mathbf{1}\}$$

$$\mathsf{STree}[\alpha, \kappa] = \oplus\{\mathbf{nd} : \mathsf{STree}[\alpha, \alpha \otimes \mathsf{STree}[\alpha, \kappa]], \mathbf{lf} : \kappa\}$$

We have that $\mathsf{Tree}[\alpha] \otimes \kappa$ is isomorphic to $\mathsf{STree}[\alpha, \kappa]$ and that the processes witnessing the isomorphism can be easily implemented (see Section 9).

At the core of type checking lies *type equality*. We show that we can translate type equality for nested session types to the trace equivalence problem for deterministic first-order grammars, shown to be decidable by Jančar, albeit with doubly-exponential complexity [31]. Solomon [42] already proved a related connection between *inductive* type equality for nested types and language equality for deterministic pushdown automata (DPDA). The difference is that the standard session type equality is defined coinductively, as a bisimulation, rather than via language equivalence [23]. This is because session types capture communication behavior rather than the structure of closed values so a type such as $\mathsf{R} = \oplus\{\mathbf{a} : \mathsf{R}\}$ is not equal to the empty type $\mathsf{E} = \oplus\{\}$. The reason is that the former type can send infinitely many $\mathbf{a}$'s while the latter cannot, and hence their communication behavior is different, implying that the types must be different. Interestingly, if we imagine a lazy functional language such as Haskell with non-generative recursive types, then $\mathsf{R}$ and $\mathsf{E}$ would also be different. In fact, nothing in our analysis of equirecursive nested types depends on linearity, just on the coinductive interpretation of types. Our key results, namely decidability of type equality and a practical algorithm for it, apply to lazy functional languages!

The decision procedure for deterministic first-order grammars does not appear to be directly suitable for implementation, in part due to its doubly-exponential complexity bound. Instead we develop an algorithm combining loop detection [23] with instantiation [18] and a special treatment of reflexivity. The algorithm is sound, but incomplete, and reports success, a counterexample, or an inconclusive outcome (which counts as failure). In our experience, the algorithm is surprisingly efficient and sufficient for all our examples.

We have implemented nested session types and integrated them with the Rast language that is based on session types [17,18,19]. We have evaluated our prototype on several examples such as the Dyck language [21], an expression server [45] and serializing binary trees, and standard polymorphic data structures such as lists, stacks and queues.

Most closely related to our work is context-free session types (CFSTs) [45]. CFSTs also enhance the expressive power of binary session types by extending types with a notion of sequential composition of types. In connection with CFSTs, we identified a proper fragment of nested session types closed under sequential composition and therefore nested session types are strictly more expressive than CFSTs.

The main technical contributions of our work are:

- A uniform language of session types supporting prenex polymorphism, type constructors, and nested types and its type safety proof (Sections 3, 6).
- A proof of decidability of type equality (Section 4).
- A practical algorithm for type equality and its soundness proof (Section 5).
- A proper fragment of nested session types that is closed under sequential composition, the main feature of context-free session types (Section 7).
- An implementation and integration with the Rast language (Section 8).

### 2 Overview of Nested Session Types

The main motivation for studying nested types is quite practical and generally applicable to programming languages with structural type systems. We start by applying parametric type constructors for a standard polymorphic queue data structure. We also demonstrate how the types can be made more precise using nesting. A natural consequence of having nested types is the ability to capture (communication) patterns characterized by context-free languages. As an illustration, we express the Dyck language of balanced parentheses and show how nested types are connected to DPDAs also.

***Queues*** A standard application of parameterized types is the definition of polymorphic data structures such as lists, stacks, or queues. As a simple example, consider the nested type:

$$\mathsf{queue}[\alpha] \triangleq \&\{\mathbf{ins} : \alpha \multimap \mathsf{queue}[\alpha], \mathbf{del} : \oplus\{\mathbf{none} : \mathbf{1}, \mathbf{some} : \alpha \otimes \mathsf{queue}[\alpha]\}\}$$

The type $\mathsf{queue}$, parameterized by $\alpha$, represents a queue with values of type $\alpha$. A process providing this type offers an *external choice* ($\&$) enabling the client to either *insert* a value of type $\alpha$ in the queue (label **ins**), or to *delete* a value from the queue (label **del**). After receiving label **ins**, the provider expects to receive a value of type $\alpha$ (the $\multimap$ operator) and then proceeds to offer $\mathsf{queue}[\alpha]$. Upon reception of the label **del**, the provider queue is either empty, in which case it sends the label **none** and terminates the session (as prescribed by type $\mathbf{1}$), or is non-empty, in which case it sends a value of type $\alpha$ (the $\otimes$ operator) and recurses with $\mathsf{queue}[\alpha]$.

Although parameterized type definitions are sufficient to express the standard interface to polymorphic data structures, we propose *nested session types* which are considerably more expressive. For instance, we can use type parameters to track the number of elements in the queue in its type!

$$\mathsf{queue}[\alpha, x] \triangleq \&\{\mathbf{ins} : \alpha \multimap \mathsf{queue}[\alpha, \mathsf{Some}[\alpha, x]], \mathbf{del} : x\}$$

$$\mathsf{Some}[\alpha, x] \triangleq \oplus\{\mathbf{some} : \alpha \otimes \mathsf{queue}[\alpha, x]\} \qquad \mathsf{None} \triangleq \oplus\{\mathbf{none} : \mathbf{1}\}$$

The second type parameter $x$ tracks the number of elements. This parameter can be understood as a *symbol stack*. On inserting an element, we recurse to $\mathsf{queue}[\alpha, \mathsf{Some}[\alpha, x]]$ denoting the *push* of $\mathsf{Some}$ symbol on stack $x$. We initiate the empty queue with the type $\mathsf{queue}[\alpha, \mathsf{None}]$ where the second parameter denotes *an empty symbol stack*. Thus, a queue with $n$ elements would have the type $\mathsf{queue}[\alpha, \mathsf{Some}^n[\alpha, \mathsf{None}]]$. On receipt of the $\mathsf{del}$ label, the type transitions to $x$ which can either be $\mathsf{None}$ (if the queue is empty) or $\mathsf{Some}[\alpha, x]$ (if the queue is non-empty). In the latter case, the type sends label **some** followed by an element, and transitions to $\mathsf{queue}[\alpha, x]$ denoting a *pop* from the symbol stack. In the former case, the type sends the label $\mathsf{none}$ and terminates. Both these behaviors are reflected in the definitions of types $\mathsf{Some}$ and $\mathsf{None}$.

***Context-Free Languages*** Recursive session types capture the class of regular languages [45]. However, in practice, many useful languages are beyond regular. As an illustration, suppose we would like to express a balanced parentheses language, also known as the Dyck language [21] with the end-marker \$. We use $\mathbf{L}$ to denote an opening symbol, and $\mathbf{R}$ to denote a closing symbol (in a session-typed mindset, $\mathbf{L}$ can represent client request and $\mathbf{R}$ is server response). We need to enforce that each $\mathbf{L}$ has a corresponding closing $\mathbf{R}$ and they are properly nested. To express this, we need to track the number of $\mathbf{L}$'s in the output with the session type. However, this notion of *memory* is beyond the expressive power of regular languages, so mere recursive session types will not suffice.

We utilize the expressive power of nested types to express this behavior.

$$T[x] \triangleq \oplus\{\mathbf{L} : T[T[x]], \mathbf{R} : x\} \qquad D \triangleq \oplus\{\mathbf{L} : T[D], \$ : \mathbf{1}\}$$

The nested type $T[x]$ takes $x$ as a type parameter and either outputs $\mathbf{L}$ and continues with $T[T[x]]$, or outputs $\mathbf{R}$ and continues with $x$. The type $D$ either outputs $\mathbf{L}$ and continues with $T[D]$, or outputs \$ and terminates. The type $D$ expresses a Dyck word with end-marker \$ [34].

The key idea here is that the number of $T$'s in the type of a word tracks the number of unmatched $\mathbf{L}$'s in it. Whenever the type $T[x]$ outputs $\mathbf{L}$, it recurses with $T[T[x]]$ incrementing the number of $T$'s in the type by 1. Dually, whenever the type outputs $\mathbf{R}$, it recurses with $x$ decrementing the number of $T$'s in the type by 1. The type $D$ denotes a balanced word with no unmatched $\mathbf{L}$'s. Moreover, since we can only output \$ (or $\mathbf{L}$) at the type $D$ and *not* $\mathbf{R}$, we obtain the invariant that any word of type $D$ must be balanced. If we imagine the parameter $x$ as the symbol stack, outputting an $\mathbf{L}$ pushes $T$ on the stack, while outputting $\mathbf{R}$ pops $T$ from the stack. The definition of $D$ ensures that once an $\mathbf{L}$ is outputted, the symbol stack is initialized with $T[D]$ indicating one unmatched $\mathbf{L}$.

Nested session types do not restrict communication so that the words represented *have to be balanced*. To this end, the type $D'$ can model the *cropped Dyck language*, where *unbalanced* words can be captured.

$$T'[x] \triangleq \oplus\{\mathbf{L} : T'[T'[x]], \mathbf{R} : x, \$ : \mathbf{1}\} \qquad D' \triangleq \oplus\{\mathbf{L} : T'[D'], \$ : \mathbf{1}\}$$

The only difference between types $T[x]$ and $T'[x]$ is that $T'[x]$ allows us to terminate at any point using the \$ label which immediately transitions to type $\mathbf{1}$. Nested session types can not only capture the class of deterministic context-free languages recognized by DPDAs that *accept by empty stack* (balanced words), but also the class of deterministic context-free languages recognized by DPDAs that *accept by final state* (cropped words).

***Multiple Kinds of Parentheses*** We can use nested types to express more general words with different kinds of parentheses. Let $\mathbf{L}$ and $\mathbf{L}'$ denote two kinds of opening symbols, while $\mathbf{R}$ and $\mathbf{R}'$ denote their corresponding closing symbols respectively. We define the session types

$$\begin{aligned} S[x] &\triangleq \oplus\{\mathbf{L} : S[S[x]], \mathbf{L}' : S'[S[x]], \mathbf{R} : x\} \\ S'[x] &\triangleq \oplus\{\mathbf{L} : S[S'[x]], \mathbf{L}' : S'[S'[x]], \mathbf{R}' : x\} \\ E &\triangleq \oplus\{\mathbf{L} : S[E], \mathbf{L}' : S'[E], \$ : \mathbf{1}\} \end{aligned}$$

We *push* symbols $S$ and $S'$ to the stack on outputting $\mathbf{L}$ and $\mathbf{L}'$ respectively. Dually, we *pop* $S$ and $S'$ from the stack on outputting $\mathbf{R}$ and $\mathbf{R}'$ respectively. Then, the type $E$ defines an *empty stack*, thereby representing a balanced Dyck word. This technique can be generalized to any number of kinds of brackets.

***Multiple States as Multiple Parameters*** Using defined type names with *multiple* type parameters, we enable types to capture the language of DPDAs with several states. Consider the language $L_3 = \{\mathbf{L}^n \mathbf{a}\, \mathbf{R}^n \mathbf{a} \cup \mathbf{L}^n \mathbf{b}\, \mathbf{R}^n \mathbf{b} \mid n > 0\}$, proposed by Korenjak and Hopcroft [34]. A word in this language starts with a sequence of opening symbols $\mathbf{L}$, followed by an *intermediate symbol*, either $\mathbf{a}$ or $\mathbf{b}$. Then, the word contains as many closing symbols $\mathbf{R}$ as there were $\mathbf{L}$s and terminates with the symbol $\mathbf{a}$ or $\mathbf{b}$ *matching* the intermediate symbol.

$$U \triangleq \oplus\{\mathbf{L} : O[C[A], C[B]]\} \qquad O[x, y] \triangleq \oplus\{\mathbf{L} : O[C[x], C[y]], \mathbf{a} : x, \mathbf{b} : y\}$$

$$C[x] \triangleq \oplus\{\mathbf{R} : x\} \qquad A \triangleq \oplus\{\mathbf{a} : \mathbf{1}\} \qquad B \triangleq \oplus\{\mathbf{b} : \mathbf{1}\}$$

The $L_3$ language is characterized by session type $U$. Since the type $U$ is unaware of which intermediate symbol among $\mathbf{a}$ or $\mathbf{b}$ would eventually be chosen, it cleverly maintains *two symbol stacks* in the two type parameters $x$ and $y$ of $O$. We initiate type $U$ with outputting **L** and transitioning to $O[C[A], C[B]]$ where the symbol $C$ tracks that we have outputted *one* **L**. The types $A$ and $B$ represent the intermediate symbols that might be used in the future. The type $O[x, y]$ can either output an **L** and transition to $O[C[x], C[y]]$ *pushing* the symbol $C$ onto *both* stacks; or it can output **a** (or **b**) and transition to the first (resp. second) type parameter $x$ (resp. $y$). Intuitively, the type parameter $x$ would have the form $C^n[A]$ for $n > 0$ (resp. $y$ would be $C^n[B]$). Then, the type $C[x]$ would output an **R** and *pop* the symbol $C$ from the stack by transitioning to $x$. Once all the closing symbols have been outputted (note that you cannot terminate pre-emptively), we transition to type $A$ or $B$ depending on the intermediate symbol chosen. Type $A$ outputs **a** and terminates, and similarly, type $B$ outputs **b** and terminates. Thus, we simulate the $L_3$ language (not possible with context-free session types [45]) using two type parameters.

More broadly, nested types can neatly capture *complex server-client interactions*. For instance, client requests can be captured using labels $\mathbf{L}, \mathbf{L}'$ while server responses can be captured using labels $\mathbf{R}, \mathbf{R}'$ expressing *multiple kinds* of requests. Balanced words will then represent that all requests have been handled. The types can also guarantee that responses do not exceed requests.

### 3 Description of Types

The underlying base system of session types is derived from a Curry-Howard interpretation [7,8] of intuitionistic linear logic [25]. Below we describe the session types, their operational interpretation and the continuation type.

| | | | | |
|---|---|---|---|---|
| $A, B, C$ | $::=$ | $\oplus\{\ell : A_\ell\}_{\ell \in L}$ | send label $k \in L$ | continue at type $A_k$ |
| | $\mid$ | $\&\{\ell : A_\ell\}_{\ell \in L}$ | receive label $k \in L$ | continue at type $A_k$ |
| | $\mid$ | $A \otimes B$ | send channel $a : A$ | continue at type $B$ |
| | $\mid$ | $A \multimap B$ | receive channel $a : A$ | continue at type $B$ |
| | $\mid$ | $\mathbf{1}$ | send close message | no continuation |
| | $\mid$ | $\alpha$ | type variable | |
| | $\mid$ | $V[\overline{B}]$ | defined type name | |

The basic type operators have the usual interpretation: the *internal choice* operator $\oplus\{\ell : A_\ell\}_{\ell \in L}$ selects a branch with label $k \in L$ with corresponding continuation type $A_k$; the *external choice* operator $\&\{\ell : A_\ell\}_{\ell \in L}$ offers a choice with labels $\ell \in L$ with corresponding continuation types $A_\ell$; the *tensor* operator $A \otimes B$ represents the channel passing type that consists of sending a channel of type $A$ and proceeding with type $B$; dually, the *lolli* operator $A \multimap B$ consists of receiving a channel of type $A$ and continuing with type $B$; the *terminated session* **1** is the operator that closes the session.

We also support *type constructors* to define new *type names*. A type name $V$ is defined according to a *type definition* $V[\overline{\alpha}] = A$ that is parameterized by a sequence of *distinct type variables* $\overline{\alpha}$ that the type $A$ can refer to. We can use type names in a type expression using $V[\overline{B}]$. Type expressions can also refer to parameter $\alpha$ available in scope. The *free variables* in type $A$ refer to the set of type variables that occur freely in $A$. Types without any free variables are called *closed types*. We call any type not of the form $V[\overline{B}]$ to be *structural*.

All type definitions are stored in a finite global *signature* $\Sigma$ defined as

$$\text{Signature } \Sigma ::= \cdot \mid \Sigma, V[\overline{\alpha}] = A$$

In a *valid signature*, all definitions $V[\overline{\alpha}] = A$ are contractive, meaning that $A$ is *structural*, i.e. not itself a type name. This allows us to take an *equirecursive* view of type definitions, which means that unfolding a type definition does not require communication. More concretely, the type $V[\overline{B}]$ is considered equivalent to its unfolding $A[\overline{B}/\overline{\alpha}]$. We can easily adapt our definitions to an *isorecursive* view [35,20] with explicit unfold messages. All type names $V$ occurring in a valid signature must be defined, and all type variables defined in a valid definition must be distinct. Furthermore, for a valid definition $V[\overline{\alpha}] = A$, the free variables occurring in $A$ must be contained in $\overline{\alpha}$. This top-level scoping of all type variables is what we call the *prenex form of polymorphism*.

## 4 Type Equality

Central to any practical type checking algorithm is type equality. In our system, it is necessary for the rule of identity (forwarding) and process spawn, as well as the channel-passing constructs for types $A \otimes B$ and $A \multimap B$. However, with nested polymorphic recursion, checking equality becomes challenging. We first develop the underlying theory of equality providing its definition, and then establish its reduction to checking trace equivalence of deterministic first-order grammars.

### 4.1 Type Equality Definition

Intuitively, two types are equal if they permit exactly the *same* communication behavior. Formally, type equality is captured using a coinductive definition following seminal work by Gay and Hole [23].

**Definition 1.** *We first define* $\mathsf{unfold}_\Sigma(A)$ *as*

$$\frac{V[\overline{\alpha}] = A \in \Sigma}{\mathsf{unfold}_\Sigma(V[\overline{B}]) = A[\overline{B}/\overline{\alpha}]}\ \mathsf{def} \qquad\qquad \frac{A \neq V[\overline{B}]}{\mathsf{unfold}_\Sigma(A) = A}\ \mathsf{str}$$

Unfolding a structural type simply returns $A$. Since type definitions are *contractive* [23], the result of unfolding is never a type name application and it always terminates in one step.

**Definition 2.** *Let Type be the set of closed type expressions (no free variables). A relation* $\mathcal{R} \subseteq \mathit{Type} \times \mathit{Type}$ *is a type bisimulation if* $(A, B) \in \mathcal{R}$ *implies:*

– *If* $\mathsf{unfold}_\Sigma(A) = \oplus\{\ell : A_\ell\}_{\ell \in L}$*, then* $\mathsf{unfold}_\Sigma(B) = \oplus\{\ell : B_\ell\}_{\ell \in L}$ *and also* $(A_\ell, B_\ell) \in \mathcal{R}$ *for all* $\ell \in L$*.*

– *If* $\mathsf{unfold}_\Sigma(A) = \&\{\ell : A_\ell\}_{\ell \in L}$*, then* $\mathsf{unfold}_\Sigma(B) = \&\{\ell : B_\ell\}_{\ell \in L}$ *and also* $(A_\ell, B_\ell) \in \mathcal{R}$ *for all* $\ell \in L$*.*
– *If* $\mathsf{unfold}_\Sigma(A) = A_1 \otimes A_2$*, then* $\mathsf{unfold}_\Sigma(B) = B_1 \otimes B_2$ *and* $(A_1, B_1) \in \mathcal{R}$ *and* $(A_2, B_2) \in \mathcal{R}$*.*
– *If* $\mathsf{unfold}_\Sigma(A) = A_1 \multimap A_2$*, then* $\mathsf{unfold}_\Sigma(B) = B_1 \multimap B_2$ *and* $(A_1, B_1) \in \mathcal{R}$ *and* $(A_2, B_2) \in \mathcal{R}$*.*
– *If* $\mathsf{unfold}_\Sigma(A) = \mathbf{1}$*, then* $\mathsf{unfold}_\Sigma(B) = \mathbf{1}$*.*

**Definition 3.** *Two closed types* $A$ *and* $B$ *are equal* ($A \equiv B$) *iff there exists a type bisimulation* $\mathcal{R}$ *such that* $(A, B) \in \mathcal{R}$*.*

When the signature $\Sigma$ is not clear from context we add a subscript, $A \equiv_\Sigma B$. This definition only applies to types with no free type variables. Since we allow parameters in type definitions, we need to define equality in the presence of free type variables. To this end, we define the notation $\forall \mathcal{V}.\, A \equiv B$ where $\mathcal{V}$ is a collection of type variables and $A$ and $B$ are valid types w.r.t. $\mathcal{V}$ (i.e., free variables in $A$ and $B$ are contained in $\mathcal{V}$).

**Definition 4.** *We define* $\forall \mathcal{V}.\, A \equiv B$ *iff for all closed type substitutions* $\sigma : \mathcal{V}$*, we have* $A[\sigma] \equiv B[\sigma]$*.*

#### 4.2 Decidability of Type Equality

Solomon [42] proved that types defined using parametric type definitions with an *inductive interpretation* can be translated to DPDAs, thus reducing type equality to language equality on DPDAs. However, our type definitions have a *coinductive interpretation.* As an example, consider the types $A = \oplus\{\mathbf{a} : A\}$ and $B = \oplus\{\mathbf{b} : B\}$. With an *inductive* interpretation, types $A$ and $B$ are empty (because they do not have terminating symbols) and, thus, are equal. However, with a *coinductive* interpretation, type $A$ will send an infinite number of $\mathbf{a}$'s, and $B$ will send an infinite number of $\mathbf{b}$'s, and are thus not equal. Our reduction needs to account for this coinductive behavior.

We show that type equality of nested session types is decidable via a reduction to the trace equivalence problem of deterministic first-order grammars [30]. A *first-order grammar* is a structure $(\mathcal{N}, \mathcal{A}, \mathcal{S})$ where $\mathcal{N}$ is a set of non-terminals, $\mathcal{A}$ is a finite set of *actions*, and $\mathcal{S}$ is a finite set of *production rules.* The arity of non-terminal $X \in \mathcal{N}$ is written as $\mathsf{arity}(X) \in \mathbb{N}$. Production rules rely on a countable set of *variables* $\mathcal{V}$, and on the set $\mathcal{T}_\mathcal{N}$ of *regular terms* over $\mathcal{N} \cup \mathcal{V}$. A term is *regular* if the set of subterms is finite (see [30]).

Each production rule has the form $X\overline{\alpha} \xrightarrow{a} E$ where $X \in \mathcal{N}$ is a non-terminal, $a \in \mathcal{A}$ is an action, and $\overline{\alpha} \in \mathcal{V}^*$ are variables that the term $E \in \mathcal{T}_\mathcal{N}$ can refer to. A grammar is *deterministic* if for each pair of $X \in \mathcal{N}$ and $a \in \mathcal{A}$, there is at most one rule of the form $X\overline{\alpha} \xrightarrow{a} E$ in $\mathcal{S}$. The substitution of terms $\overline{B}$ for variables $\overline{\alpha}$ in a rule $X\overline{\alpha} \xrightarrow{a} E$, denoted by $X\overline{B} \xrightarrow{a} E[\overline{B}/\overline{\alpha}]$, is the rule $(X\overline{\alpha} \xrightarrow{a} E)[\overline{B}/\overline{\alpha}]$. Given a set of rules $\mathcal{S}$, the trace of a term $T$ is defined as $\mathsf{traces}_\mathcal{S}(T) = \{\overline{a} \in \mathcal{A}^* \mid (T \xrightarrow{\overline{a}} T') \in \mathcal{S},\ \text{for some } T' \in \mathcal{T}_\mathcal{N}\}$. Two terms are *trace equivalent*, written as $T \sim_\mathcal{S} T'$, if $\mathsf{traces}_\mathcal{S}(T) = \mathsf{traces}_\mathcal{S}(T')$.

The crux of the reduction lies in the observation that session types can be translated to terms and type definitions can be translated to production rules of a first-order grammar. We start the translation of nested session types to grammars by first making an initial pass over the signature and introducing fresh *internal names* such that the new type definitions alternate between structural (except $\mathbf{1}$ and $\alpha$) and non-structural types. These internal names are parameterized over their free type variables, and their definitions are added to the signature. This *internal renaming* simplifies the next step where we translate this extended signature to grammar production rules.

*Example 1.* As a running example, consider the queue type from Section 2:

$$Q[\alpha] = \&\{\mathbf{ins} : \alpha \multimap Q[\alpha], \mathbf{del} : \oplus\{\mathbf{none} : \mathbf{1}, \mathbf{some} : \alpha \otimes Q[\alpha]\}\}$$

After performing internal renaming for this type, we obtain the following signature:

$$\begin{array}{ll} Q[\alpha] = \&\{\mathbf{ins} : X_0[\alpha], \mathbf{del} : X_1[\alpha]\} & X_1[\alpha] = \oplus\{\mathbf{none} : \mathbf{1}, \mathbf{some} : X_2[\alpha]\} \\ X_0[\alpha] = \alpha \multimap Q[\alpha] & X_2[\alpha] = \alpha \otimes Q[\alpha] \end{array}$$

We introduce the fresh internal names $X_0$, $X_1$ and $X_2$ (parameterized with free variable $\alpha$) to represent the continuation type in each case. Note the alternation between structural and non-structural types (of the form $V[\overline{B}]$).

Next, we translate this extended signature to the grammar $\mathcal{G} = (\mathcal{N}, \mathcal{A}, \mathcal{S})$ aimed at reproducing the behavior prescribed by the types as grammar actions.

$$\begin{aligned} \mathcal{N} &= \{Q, X_0, X_1, X_2, \bot\} \\ \mathcal{A} &= \{\&\mathbf{ins}, \&\mathbf{del}, \multimap_1, \multimap_2\ \oplus\mathbf{none}, \oplus\mathbf{some}, \otimes_1, \otimes_2, \} \\ \mathcal{S} &= \{Q\alpha \xrightarrow{\&\mathbf{ins}} X_0\alpha,\ Q\alpha \xrightarrow{\&\mathbf{del}} X_1\alpha,\ X_0\alpha \xrightarrow{\multimap_1} \alpha,\ X_0\alpha \xrightarrow{\multimap_2} Q\alpha, \\ &\qquad X_1\alpha \xrightarrow{\oplus\mathbf{none}} \bot,\ X_1\alpha \xrightarrow{\oplus\mathbf{some}} X_2\alpha,\ X_2\alpha \xrightarrow{\otimes_1} \alpha,\ X_2\alpha \xrightarrow{\otimes_2} Q\alpha\} \end{aligned}$$

Essentially, each defined type name is translated to a fresh non-terminal. Each type definition then corresponds a sequence of rules: one for each possible continuation type with the appropriate label that leads to that continuation. For instance, the type $Q[\alpha]$ has two possible continuations: transition to $X_0[\alpha]$ with action $\&\mathbf{ins}$ or to $X_1[\alpha]$ with action $\&\mathbf{del}$. The rules for all other type names is analogous. When the continuation is $\mathbf{1}$, we transition to the nullary non-terminal $\bot$ disabling any further action. When the continuation is $\alpha$, we transition to $\alpha$. Since each type name is defined once, the produced grammar is deterministic.

Formally, the translation from an (extended) signature to a grammar is handled by two simultaneous tasks: translating type definitions into production rules (function $\tau$ below), and converting type names, variables and the terminated session into grammar terms (function $(\!|\cdot|\!)$). The function $(\!|\cdot|\!) : \mathit{OType} \to \mathcal{T}_{\mathcal{N}}$ from open session types to grammar terms is defined by:

$$\begin{array}{rll} (\!|\mathbf{1}|\!) &= \bot & \text{type } \mathbf{1} \text{ translates to } \bot \\ (\!|\alpha|\!) &= \alpha & \text{type variables translate to themselves} \\ (\!|V[B_1, \ldots, B_n]|\!) &= V (\!|B_1|\!) \cdots (\!|B_n|\!) & \text{type names translate to first-order terms} \end{array}$$

Due to this mapping, throughout this section we will use type names indistinctly as type names or as non-terminal first-order symbols.

The function $\tau$ converts a type definition $V[\overline{\alpha}] = A$ into a set of production rules and is defined according to the structure of $A$ as follows:

$$\begin{aligned}
\tau(V[\overline{\alpha}] = \oplus\{\ell\colon A_\ell\}_{\ell\in L}) &= \{(\!|V[\overline{\alpha}]|\!) \xrightarrow{\oplus\ell} (\!|A_\ell|\!) \mid \ell \in L\}\\
\tau(V[\overline{\alpha}] = \&\{\ell\colon A_\ell\}_{\ell\in L}) &= \{(\!|V[\overline{\alpha}]|\!) \xrightarrow{\&\ell} (\!|A_\ell|\!) \mid \ell \in L\}\\
\tau(V[\overline{\alpha}] = A_1 \otimes A_2) &= \{(\!|V[\overline{\alpha}]|\!) \xrightarrow{\otimes_i} (\!|A_i|\!) \mid i = 1, 2\}\\
\tau(V[\overline{\alpha}] = A_1 \multimap A_2) &= \{(\!|V[\overline{\alpha}]|\!) \xrightarrow{\multimap_i} (\!|A_i|\!) \mid i = 1, 2\}
\end{aligned}$$

The function $\tau$ identifies the actions and continuation types corresponding to $A$ and translates them to grammar rules. Internal and external choices lead to actions $\oplus\ell$ and $\&\ell$, for each $\ell \in L$, with $A_\ell$ as the continuation type. The type $A_1 \otimes A_2$ enables two possible actions, $\otimes_1$ and $\otimes_2$, with continuation $A_1$ and $A_2$ respectively. Similarly $A_1 \multimap A_2$ produces the actions $\multimap_1$ and $\multimap_2$ with $A_1$ and $A_2$ as respective continuations. Contractiveness ensures that there are no definitions of the form $V[\overline{\alpha}] = V'[\overline{B}]$. Our internal renaming ensures that we do not encounter cases of the form $V[\overline{\alpha}] = \mathbf{1}$ or $V[\overline{\alpha}] = \alpha$ because we do not generate internal names for them. For the same reason, the $(\!|\cdot|\!)$ function is only defined on the complement types $\mathbf{1}$, $\alpha$ and $V[\overline{B}]$.

The $\tau$ function is extended to translate a signature by being applied pointwise. Formally, $\tau(\Sigma) = \bigcup_{(V[\overline{\alpha}]=A)\in\Sigma} \tau(V[\overline{\alpha}] = A)$. Connecting all pieces, we define the $\mathsf{fog}$ function that translates a signature to a grammar as:

$$\begin{aligned}
&\mathsf{fog}(\Sigma) = (\mathcal{N}, \mathcal{A}, \mathcal{S}), \text{ where:} && \mathcal{S} = \tau(\Sigma)\\
&\mathcal{N} = \{X \mid (X\overline{\alpha} \xrightarrow{a} E) \in \tau(\Sigma)\} && \mathcal{A} = \{a \mid (X\overline{\alpha} \xrightarrow{a} E) \in \tau(\Sigma)\}
\end{aligned}$$

The grammar is constructed by first computing $\tau(\Sigma)$ to obtain all the production rules. $\mathcal{N}$ and $\mathcal{A}$ are constructed by collecting the set of non-terminals and actions from these rules. The finite representation of session types and uniqueness of definitions ensure that $\mathsf{fog}(\Sigma)$ is a deterministic first-order grammar.

Checking equality of types $A$ and $B$ given signature $\Sigma$ finally reduces to (i) internal renaming of $\Sigma$ to produce $\Sigma'$, and (ii) checking trace-equivalence of terms $(\!|A|\!)$ and $(\!|B|\!)$ given grammar $\mathsf{fog}(\Sigma')$. If $A$ and $B$ are themselves structural, we generate internal names for them also during the internal renaming process. Since we assume an *equirecursive* and *non-generative* view of types, it is easy to show that internal renaming does not alter the communication behavior of types and preserves type equality. Formally, $A \equiv_\Sigma B$ iff $A \equiv_{\Sigma'} B$.

**Theorem 1.** *$A \equiv_\Sigma B$ if and only if $(\!|A|\!) \sim_\mathcal{S} (\!|B|\!)$, where $(\mathcal{N}, \mathcal{A}, \mathcal{S}) = \mathsf{fog}(\Sigma')$ and $\Sigma'$ is the extended signature for $\Sigma$.*

*Proof.* For the direct implication, assume that $(\!|A|\!) \not\sim_\mathcal{S} (\!|B|\!)$. Pick a sequence of actions in the difference of the traces and let $w_0$ be its greatest prefix occurring in both traces. Either $w_0$ is a maximal trace for one of the terms, or we have $(\!|A|\!) \xrightarrow{w_0} (\!|A'|\!)$ and $(\!|B|\!) \xrightarrow{w_0} (\!|B'|\!)$, with $(\!|A'|\!) \xrightarrow{a_1} (\!|A''|\!)$ and $(\!|B'|\!) \xrightarrow{a_2} (\!|B''|\!)$, where

$a_1 \not= a_2$. In both cases, with a simple case analysis on the definition of the translation $\tau$, we conclude that $A' \not\equiv B'$ and so $A \not\equiv B$. For the reciprocal implication, assume that $(\!|A|\!) \sim_{\mathcal{S}} (\!|B|\!)$. Consider the relation

$$\mathcal{R} = \{(A_0, B_0) \mid \mathsf{trace}_{\mathcal{S}}((\!|A_0|\!)) = \mathsf{trace}_{\mathcal{S}}((\!|B_0|\!))\} \subseteq \mathit{Type} \times \mathit{Type}.$$

Obviously, $(A, B) \in \mathcal{R}$. To prove that $\mathcal{R}$ is a type bisimulation, let $(A_0, B_0) \in \mathcal{R}$ and proceed by case analysis on $A_0$ and $B_0$. For the case $\mathsf{unfold}_{\Sigma}(A_0) = \oplus\{\ell\colon A_\ell\}_{\ell \in L}$, we have $(\!|A_0|\!) \xrightarrow{\oplus\ell} (\!|A_\ell|\!)$. Since, by hypothesis, the traces coincide, $\mathsf{trace}_{\mathcal{S}}((\!|A_0|\!)) = \mathsf{trace}_{\mathcal{S}}((\!|B_0|\!))$, we have $(\!|B_0|\!) \xrightarrow{\oplus\ell} (\!|B_\ell|\!)$ and, thus, $\mathsf{unfold}_{\Sigma}(B_0) = \oplus\{\ell\colon B_\ell\}_{\ell \in L}$. Moreover, Jančar [30] proves that $\mathsf{trace}_{\mathcal{S}}((\!|A_\ell|\!)) = \mathsf{trace}_{\mathcal{S}}((\!|B_\ell|\!))$. Hence, $(A_\ell, B_\ell) \in \mathcal{R}$. The other cases and a detailed proof can be found in [14].

However, type equality is not only restricted to closed types (see Definition 4). To decide equality for open types, i.e. $\forall \mathcal{V}.\, A \equiv B$ given signature $\Sigma$, we introduce a fresh label $\ell_\alpha$ and type $A_\alpha$ for each $\alpha \in \mathcal{V}$. We extend the signature with type definitions: $\Sigma^* = \Sigma \cup_{\alpha \in \mathcal{V}} \{A_\alpha = \oplus\{\ell_\alpha\colon A_\alpha\}\}$. We then replace all occurrences of $\alpha$ in $A$ and $B$ with $A_\alpha$ and check their equality with signature $\Sigma^*$. We prove that this substitution preserves equality.

**Theorem 2.** *$\forall \mathcal{V}.\, A \equiv_{\Sigma} B$ iff $A[\sigma^*] \equiv_{\Sigma^*} B[\sigma^*]$ where $\sigma^*(\alpha) = A_\alpha$ for all $\alpha \in \mathcal{V}$.*

*Proof (Sketch).* The direct implication is trivial since $\sigma^*$ is a closed substitution. Reciprocally, we assume that $\forall \mathcal{V}.\, A \not\equiv_{\Sigma} B$. Then there must exist some substitution $\sigma'$ such that $A[\sigma'] \not\equiv_{\Sigma} B[\sigma']$. We use this constraint to and prove that $A[\sigma^*] \not\equiv_{\Sigma^*} B[\sigma^*]$. The exact details can be found in our tech report [14].

**Theorem 3.** *Checking $\forall \mathcal{V}.\, A \equiv B$ is decidable.*

*Proof.* Theorem 2 reduces equality of open types to equality of closed types. Theorem 1 reduces equality of closed nested session types to trace equivalence of first-order grammars. Jančar [30] proved that trace equivalence for first-order grammars is decidable, hence establishing the decidability of equality for nested session types.

### 5 Practical Algorithm for Type Equality

Although type equality can be reduced to trace equivalence for first-order grammars (Theorem 1 and Theorem 2), the latter problem has a very high theoretical complexity with no known practical algorithm [30]. In response, we have designed a coinductive algorithm for approximating type equality. Taking inspiration from Gay and Hole [23], we attempt to construct a bisimulation. Our proposed algorithm is sound but incomplete and can terminate in three states: *(i)* types are proved equal by constructing a bisimulation, *(ii)* counterexample detected by identifying a position where types differ, or *(iii)* terminated without a conclusive answer due to incompleteness. We interpret both *(ii)* and *(iii)* as a failure of type-checking (but there is a recourse; see Section 5.1). The algorithm

$$\frac{\mathcal{V}\ ;\ \Gamma \vdash A_\ell \equiv B_\ell \quad (\forall \ell \in L)}{\mathcal{V}\ ;\ \Gamma \vdash \oplus\{\ell : A_\ell\}_{\ell \in L} \equiv \oplus\{\ell : B_\ell\}_{\ell \in L}}\ \oplus \qquad \frac{\mathcal{V}\ ;\ \Gamma \vdash A_\ell \equiv B_\ell \quad (\forall \ell \in L)}{\mathcal{V}\ ;\ \Gamma \vdash \&\{\ell : A_\ell\}_{\ell \in L} \equiv \&\{\ell : B_\ell\}_{\ell \in L}}\ \&$$

$$\frac{\mathcal{V}\ ;\ \Gamma \vdash A_1 \equiv B_1 \quad \mathcal{V}\ ;\ \Gamma \vdash A_2 \equiv B_2}{\mathcal{V}\ ;\ \Gamma \vdash A_1 \otimes A_2 \equiv B_1 \otimes B_2}\ \otimes \qquad \frac{\mathcal{V}\ ;\ \Gamma \vdash A_1 \equiv B_1 \quad \mathcal{V}\ ;\ \Gamma \vdash A_2 \equiv B_2}{\mathcal{V}\ ;\ \Gamma \vdash A_1 \multimap A_2 \equiv B_1 \multimap B_2}\ \multimap$$

$$\frac{}{\mathcal{V}\ ;\ \Gamma \vdash \mathbf{1} \equiv \mathbf{1}}\ \mathbf{1} \qquad \frac{\alpha \in \mathcal{V}}{\mathcal{V}\ ;\ \Gamma \vdash \alpha \equiv \alpha}\ \mathsf{var} \qquad \frac{\mathcal{V}\ ;\ \Gamma \vdash \overline{A} \equiv \overline{A'}}{\mathcal{V}\ ;\ \Gamma \vdash V[\overline{A}] \equiv V[\overline{A'}]}\ \mathsf{refl}$$

$$\frac{\begin{array}{c} V_1[\overline{\alpha_1}] = A \in \Sigma \qquad V_2[\overline{\alpha_2}] = B \in \Sigma \qquad \mathcal{C} = \langle \mathcal{V}\ ;\ V_1[\overline{A_1}] \equiv V_2[\overline{A_2}] \rangle \\ \mathcal{V}\ ;\ \Gamma, \mathcal{C} \vdash_\Sigma A[\overline{A_1}/\overline{\alpha_1}] \equiv B[\overline{A_2}/\overline{\alpha_2}] \end{array}}{\mathcal{V}\ ;\ \Gamma \vdash_\Sigma V_1[\overline{A_1}] \equiv V_2[\overline{A_2}]}\ \mathsf{expd}$$

$$\frac{\begin{array}{c} \langle \mathcal{V}'\ ;\ V_1[\overline{A_1'}] \equiv V_2[\overline{A_2'}] \rangle \in \Gamma \\ \exists \sigma' : \mathcal{V}'.\ \left(\mathcal{V}\ ;\ \Gamma \Vdash V_1[\overline{A_1'[\sigma']}] \equiv V_1[\overline{A_1}] \wedge \mathcal{V}\ ;\ \Gamma \Vdash V_2[\overline{A_2'[\sigma']}] \equiv V_2[\overline{A_2}]\right) \end{array}}{\mathcal{V}\ ;\ \Gamma \vdash V_1[\overline{A_1}] \equiv V_2[\overline{A_2}]}\ \mathsf{def}$$

**Fig. 1.** Algorithmic Rules for Type Equality is deterministic (no backtracking) and the implementation is quite efficient in practice. For all our examples, type checking is instantaneous (see Section 8).

The fundamental operation in the equality algorithm is *loop detection* where we determine if we have already added an equation $A \equiv B$ to the bisimulation we are constructing. Due to the presence of *open types* with free type variables, determining if we have considered an equation already becomes a difficult operation. To that purpose, we make an initial pass over the given types and introduce fresh internal names as described in Example 1 (but also for $\mathbf{1}$ and $\alpha$ for simplicity). In the resulting signature defined type names and structural types alternate and we can perform loop detection entirely on defined type names (whether internal or external). The formal rules for this internal renaming are described in the technical report [14].

Based on the invariants established by internal names, the algorithm only needs to alternately compare two type names or two *structural types*. The rules are shown in Figure 1. The judgment has the form $\mathcal{V}\ ;\ \Gamma \vdash_\Sigma A \equiv B$ where $\mathcal{V}$ contains the free type variables in the types $A$ and $B$, $\Sigma$ is a fixed *valid* signature containing type definitions of the form $V[\overline{\alpha}] = C$, and $\Gamma$ is a collection of *closures* $\langle \mathcal{V}\ ;\ V_1[\overline{A_1}] \equiv V_2[\overline{A_2}] \rangle$. If a derivation can be constructed, all *closed instances* of all closures are included in the resulting bisimulation (see the proof of Theorem 4). A closed instance of closure $\langle \mathcal{V}\ ;\ V_1[\overline{A_1}] \equiv V_2[\overline{A_2}] \rangle$ is obtained by applying a closed substitution $\sigma$ over variables in $\mathcal{V}$, i.e., $V_1[\overline{A_1[\sigma]}] \equiv V_2[\overline{A_2[\sigma]}]$ such that the types $V_1[\overline{A_1[\sigma]}]$ and $V_2[\overline{A_2[\sigma]}]$ have no free type variables. Because the signature $\Sigma$ is fixed, we elide it from the rules in Figure 1.

In the type equality algorithm, the rules for type operators simply compare the components. If the type constructors (or the label sets in the $\oplus$ and $\&$ rules)

do not match, then type equality fails having constructed a counterexample to bisimulation. Similarly, two type variables are considered equal iff they have the same name, as exemplified by the var rule.

The rule of reflexivity is needed explicitly here (but not in the version of Gay and Hole) due to the incompleteness of the algorithm: we may otherwise fail to recognize type names parameterized with equal types as equal. Note that the refl rule checks a sequence of types in the premise.

Now we come to the key rules, expd and def. In the expd rule we expand the definitions of $V_1[\overline{A_1}]$ and $V_2[\overline{A_2}]$, and add the closure $\langle \mathcal{V} \; ; \; V_1[\overline{A_1}] \equiv V_2[\overline{A_2}]\rangle$ to $\Gamma$. Since the equality of $V_1[\overline{A_1}]$ and $V_2[\overline{A_2}]$ must hold for all its closed instances, the extension of $\Gamma$ with the corresponding closure remembers exactly that.

The def rule only applies when there already exists a closure in $\Gamma$ with the same type names $V_1$ and $V_2$. In that case, we try to find a substitution $\sigma'$ over $\mathcal{V}'$ such that $V_1[\overline{A_1}]$ is equal to $V_1[\overline{A_1'}[\sigma']]$ and $V_2[\overline{A_2}]$ is equal to $V_2[\overline{A_2'}[\sigma']]$. Immediately after, the refl rule applies and recursively calls the equality algorithm on both type parameters. The substitution $\sigma'$ is computed by a standard matching algorithm on first-order terms (which is linear-time), applied on the syntactic structure of the types. Existence of such a substitution ensures that any closed instance of $\langle \mathcal{V} \; ; \; V_1[\overline{A_1}] \equiv V_2[\overline{A_2}]\rangle$ is also a closed instance of $\langle \mathcal{V}' \; ; \; V_1[\overline{A_1'}] \equiv V_2[\overline{A_2'}]\rangle$, which are already present in the constructed type bisimulation, and we can terminate our equality check, having successfully *detected a loop.*

The algorithm so far is sound, but potentially non-terminating. There are two points of non-termination: *(i)* when encountering name/name equations, we can use the expd rule indefinitely, and *(ii)* we call the type equality recursively in the def rule. To ensure termination in the former case, we restrict the expd rule so that for any pair of type names $V_1$ and $V_2$ there is an upper bound on the number of closures of the form $\langle - \; ; \; V_1[-] \equiv V_2[-]\rangle$ allowed in $\Gamma$. We define this upper bound as the *depth bound* of the algorithm and allow the programmer to specify this depth bound. Surprisingly, a depth bound of 1 suffices for all of our examples. In the latter case, instead of calling the general type equality algorithm, we introduce the notion of *rigid equality*, denoted by $\mathcal{V} \; ; \; \Gamma \Vdash A \equiv B$. The only difference between general and rigid equality is that we cannot employ the expd rule for rigid equality. Since the size of the types reduce in all equality rules except for expd, this algorithm terminates. When comparing two instantiated type names, our algorithm first tries reflexivity, then tries to close a loop with def, and only if neither of these is applicable or fails do we expand the definitions with the expd rule. Note that if type names have no parameters, our algorithm specializes to Gay and Hole's (with the small optimizations of reflexivity and internal naming), which means our algorithm is sound and complete on monomorphic types.

***Soundness.*** We establish the soundness of the equality algorithm by constructing a type bisimulation from a derivation of $\mathcal{V} \; ; \; \Gamma \vdash A \equiv B$ by *(i)* collecting the conclusions of all the sequents, and *(ii)* forming all closed instances from them.

**Definition 5.** *Given a derivation $\mathcal{D}$ of $\mathcal{V}\ ;\ \Gamma \vdash A \equiv B$, we define the set $\mathcal{S}(\mathcal{D})$ of closures. For each sequent (regular or rigid) of the form $\mathcal{V}\ ;\ \Gamma \vdash A \equiv B$ in $\mathcal{D}$, we include the closure $\langle \mathcal{V}\ ;\ A \equiv B\rangle$ in $\mathcal{S}(\mathcal{D})$.*

**Theorem 4 (Soundness).** *If $\mathcal{V}\ ;\ \cdot \vdash A \equiv B$, then $\forall \mathcal{V}.\, A \equiv B$. Consequently, if $\mathcal{V}$ is empty, we get $A \equiv B$.*

*Proof.* Given a derivation $\mathcal{D}_0$ of $\mathcal{V}_0\ ;\ \cdot \vdash A_0 \equiv B_0$, construct $\mathcal{S}(\mathcal{D}_0)$ and define relation $\mathcal{R}_0$ as follows:

$$\mathcal{R}_0 = \{(A[\sigma], B[\sigma]) \mid \langle \mathcal{V}\ ;\ A \equiv B\rangle \in \mathcal{S}(\mathcal{D}_0) \text{ and } \sigma \text{ over } \mathcal{V}\}$$

Then, construct $\mathcal{R}_i$ ($i \geq 1$) as follows:

$$\mathcal{R}_i = \{(V[\overline{A}], V[\overline{B}]) \mid V[\overline{\alpha}] = C \in \Sigma \text{ and } (A^j, B^j) \in \mathcal{R}_{i-1}\ \forall j \in 1..|\overline{\alpha}|\}$$

Consider $\mathcal{R}$ to be the *reflexive transitive closure* of $\bigcup_{i\geq 0} \mathcal{R}_i$. Note that extending a relation by its reflexive transitive closure preserves its bisimulation properties since the bisimulation is strong. If $\mathcal{R}$ is a type bisimulation, then our theorem follows since the closure $\langle \mathcal{V}_0\ ;\ A_0 \equiv B_0\rangle \in \mathcal{S}(\mathcal{D}_0)$, and hence, for any closed substitution $\sigma$, $(A_0[\sigma], B_0[\sigma]) \in \mathcal{R}$.

All that remains is to prove that $\mathcal{R}$ is a type bisimulation. We achive this via a case analysis on the rule that added a pair $(A, B)$ to $\mathcal{R}$. The complete proof is described in the technical report [14].

#### 5.1 Type Equality Declarations

One of the primary sources of incompleteness in our algorithm is its inability to *generalize the coinductive hypothesis*. As an illustration, consider the following two types $D$ and $D'$, which only differ in the names, but have the same structure.

$$T[x] \triangleq \oplus\{\mathbf{L} : T[T[x]], \mathbf{R} : x\} \qquad D \triangleq \oplus\{\mathbf{L} : T[D], \$ : \mathbf{1}\}$$

$$T'[x] \triangleq \oplus\{\mathbf{L} : T'[T'[x]], \mathbf{R} : x\} \qquad D' \triangleq \oplus\{\mathbf{L} : T'[D'], \$ : \mathbf{1}\}$$

To establish $D \equiv D'$, our algorithm explores the $\mathbf{L}$ branch and checks $T[D] \equiv T'[D']$. A corresponding closure $\langle \cdot\ ;\ T[D] \equiv T'[D']\rangle$ is added to $\Gamma$, and our algorithm then checks $T[T[D]] \equiv T'[T'[D']]$. This process repeats until it exceeds the depth bound and terminates with an inconclusive answer. What the algorithm never realizes is that $T[x] \equiv T'[x]$ for all $x \in \mathit{Type}$; it fails to generalize to this hypothesis and is always inserting closed equality constraints to $\Gamma$.

To allow a recourse, we permit the programmer to declare (concrete syntax)

```
eqtype T[x] = T'[x]
```

an equality constraint easily verified by our algorithm. We then *seed* the $\Gamma$ in the equality algorithm with the corresponding closure from the `eqtype` constraint which can then be used to establish $D \equiv D'$

$$\cdot\ ;\ \langle x\ ;\ T[x] \equiv T'[x]\rangle \vdash D \equiv D'$$

which, upon exploring the **L** branch reduces to

$$\cdot \; ; \; \langle x \; ; \; T[x] \equiv T'[x] \rangle, \langle \cdot \; ; \; D \equiv D' \rangle \vdash T[D] \equiv T'[D']$$

which holds under the substitution $[D/x]$ as required by the def rule.

In the implementation, we first collect all the eqtype declarations in the program into a global set of closures $\Gamma_0$. We then validate every eqtype declaration by checking $\mathcal{V} \; ; \; \Gamma_0 \vdash A \equiv B$ for every pair $(A, B)$ (with free variables $\mathcal{V}$) in the eqtype declarations. Essentially, this ensures that all equality declarations are valid w.r.t. each other. Finally, all equality checks are then performed under this more general $\Gamma_0$. The soundness of this approach can be proved with the following more general theorem.

**Theorem 5 (Seeded Soundness).** *For a valid set of* eqtype *declarations* $\Gamma_0$, *if* $\mathcal{V} \; ; \; \Gamma_0 \vdash A \equiv B$, *then* $\forall \mathcal{V}. \, A \equiv B$.

Our soundness proof can easily be modified to accommodate this requirement. Intuitively, since $\Gamma_0$ is valid, all closed instances of $\Gamma_0$ are already proven to be bisimilar. Thus, all properties of a type bisimulation are still preserved if all closed instances of $\Gamma_0$ are added to it.

One final note on the rule of reflexivity: a type name may *not* actually depend on its parameter. As a simple example, we have $V[\alpha] = \mathbf{1}$; a more complicated one would be $V[\alpha] = \oplus\{a : V[V[\alpha]], b : \mathbf{1}\}$. When applying reflexivity, we would like to conclude that $V[A] \equiv V[B]$ regardless of $A$ and $B$. This could be easily established with an equality type declaration eqtype $V[\alpha] = V[\beta]$. In order to avoid this syntactic overhead for the programmer, we determine for each parameter $\alpha$ of each type name $V$ whether its definition is nonvariant in $\alpha$. This information is recorded in the signature and used when applying the reflexivity rule by ignoring nonvariant arguments.

### 6 Formal Language Description

In this section, we present the program constructs we have designed to realize nested polymorphism which have also been integrated with the Rast language [17,18,19] to support general-purpose programming. The underlying base system of session types is derived from a Curry-Howard interpretation [7,8] of intuitionistic linear logic [25]. The key idea is that an intuitionistic linear sequent $A_1 \; A_2 \; \ldots \; A_n \vdash A$ is interpreted as the interface to a process $P$. We label each of the antecedents with a channel name $x_i$ and the succedent with channel name $z$. The $x_i$'s are *channels used by* $P$ and $z$ is the *channel provided by* $P$.

$$(x_1 : A_1) \; (x_2 : A_2) \ldots (x_n : A_n) \vdash P :: (z : C)$$

The resulting judgment formally states that process $P$ provides a service of session type $C$ along channel $z$, while using the services of session types $A_1, \ldots, A_n$ provided along channels $x_1, \ldots, x_n$ respectively. All these channels must be distinct. We abbreviate the antecedent of the sequent by $\Delta$.

Due to the presence of type variables, the formal typing judgment is extended with $\mathcal{V}$ and written as

| Type | Cont. | Process Term | Cont. | Description |
|---|---|---|---|---|
| $c : \oplus\{\ell : A_\ell\}_{\ell \in L}$ | $c : A_k$ | $c.k \;;\; P$ | $P$ | send label $k$ on $c$ |
| | | $\mathsf{case}\; c\; (\ell \Rightarrow Q_\ell)_{\ell \in L}$ | $Q_k$ | receive label $k$ on $c$ |
| $c : \&\{\ell : A_\ell\}_{\ell \in L}$ | $c : A_k$ | $\mathsf{case}\; c\; (\ell \Rightarrow P_\ell)_{\ell \in L}$ | $P_k$ | receive label $k$ on $c$ |
| | | $c.k \;;\; Q$ | $Q$ | send label $k$ on $c$ |
| $c : A \otimes B$ | $c : B$ | $\mathsf{send}\; c\; w \;;\; P$ | $P$ | send channel $w : A$ on $c$ |
| | | $y \leftarrow \mathsf{recv}\; c \;;\; Q_y$ | $Q_y[w/y]$ | receive channel $w : A$ on $c$ |
| $c : A \multimap B$ | $c : B$ | $y \leftarrow \mathsf{recv}\; c \;;\; P_y$ | $P_y[w/y]$ | receive channel $w : A$ on $c$ |
| | | $\mathsf{send}\; c\; w \;;\; Q$ | $Q$ | send channel $w : A$ on $c$ |
| $c : \mathbf{1}$ | — | $\mathsf{close}\; c$ | — | send *close* on $c$ |
| | | $\mathsf{wait}\; c \;;\; Q$ | $Q$ | receive *close* on $c$ |

**Table 1.** Session types with operational description

$$\mathcal{V} \;;\; \Delta \vdash_\Sigma P :: (x : A)$$

where $\mathcal{V}$ stores the type variables $\alpha$, $\Delta$ represents the linear antecedents $x_i : A_i$, $P$ is the process expression and $x : A$ is the linear succedent. We propose and maintain that all free type variables in $\Delta, P$, and $A$ are contained in $\mathcal{V}$. Finally, $\Sigma$ is a fixed valid signature containing type and process definitions. Table 1 overviews the session types, their associated process terms, their continuation (both in types and terms) and operational description. For each type, the first line describes the provider's viewpoint, while the second line describes the client's matching but dual viewpoint.

We formalize the operational semantics as a system of *multiset rewriting rules* [9]. We introduce semantic objects $\mathsf{proc}(c, P)$ and $\mathsf{msg}(c, M)$ which mean that process $P$ or message $M$ provide along channel $c$. A process configuration is a multiset of such objects, where any two provided channels are distinct.

### 6.1 Basic Session Types

We briefly review the structural types already existing in the Rast language. The *internal choice* type constructor $\oplus\{\ell : A_\ell\}_{\ell \in L}$ is an $n$-ary labeled generalization of the additive disjunction $A \oplus B$. Operationally, it requires the provider of $x : \oplus\{\ell : A_\ell\}_{\ell \in L}$ to send a label label $k \in L$ on channel $x$ and continue to provide type $A_k$. The corresponding process term is written as $(x.k \;;\; P)$ where the continuation $P$ provides type $x : A_k$. Dually, the client must branch based on the label received on $x$ using the process term $\mathsf{case}\; x\; (\ell \Rightarrow Q_\ell)_{\ell \in L}$ where $Q_\ell$ is the continuation in the $\ell$-th branch.

$$\frac{(k \in L) \quad \mathcal{V} \;;\; \Delta \vdash P :: (x : A_k)}{\mathcal{V} \;;\; \Delta \vdash (x.k \;;\; P) :: (x : \oplus\{\ell : A_\ell\}_{\ell \in L})} \oplus R$$

$$\frac{(\forall \ell \in L) \quad \mathcal{V} \;;\; \Delta, (x : A_\ell) \vdash Q_\ell :: (z : C)}{\mathcal{V} \;;\; \Delta, (x : \oplus\{\ell : A_\ell\}_{\ell \in L}) \vdash \mathsf{case}\; x\; (\ell \Rightarrow Q_\ell)_{\ell \in L} :: (z : C)} \oplus L$$

Communication is asynchronous, so that the client $(c.k\ ;\ Q)$ sends a message $k$ along $c$ and continues as $Q$ without waiting for it to be received. As a technical device to ensure that consecutive messages on a channel arrive in order, the sender also creates a fresh continuation channel $c'$ so that the message $k$ is actually represented as $(c.k\ ;\ c \leftrightarrow c')$ (read: send $k$ along $c$ and continue along $c'$). When the message $k$ is received along $c$, we select branch $k$ and also substitute the continuation channel $c'$ for $c$.

$$(\oplus S) : \mathsf{proc}(c, c.k\ ;\ P) \ \mapsto\ \mathsf{proc}(c', P[c'/c]), \mathsf{msg}(c, c.k\ ;\ c \leftrightarrow c')$$
$$(\oplus C) : \mathsf{msg}(c, c.k\ ;\ c \leftrightarrow c'), \mathsf{proc}(d, \mathsf{case}\ c\ (\ell \Rightarrow Q_\ell)_{\ell \in L}) \ \mapsto\ \mathsf{proc}(d, Q_k[c'/c])$$

The *external choice* constructor $\&\{\ell : A_\ell\}_{\ell \in L}$ generalizes additive conjunction and is the *dual* of internal choice reversing the role of the provider and client. The corresponding rules for statics and dynamics are skipped for brevity and presented in the technical report [14].

The *tensor* operator $A \otimes B$ prescribes that the provider of $x : A \otimes B$ sends a channel, say $w$ of type $A$ and continues to provide type $B$. The corresponding process term is $\mathsf{send}\ x\ w\ ;\ P$ where $P$ is the continuation. Correspondingly, its client must receive a channel on $x$ using the term $y \leftarrow \mathsf{recv}\ x\ ;\ Q$, binding it to variable $y$ and continuing to execute $Q$.

$$\frac{\mathcal{V}\ ;\ \Delta \vdash P :: (x : B)}{\mathcal{V}\ ;\ \Delta, (y : A) \vdash (\mathsf{send}\ x\ y\ ;\ P) :: (x : A \otimes B)}\ \otimes R$$

$$\frac{\mathcal{V}\ ;\ \Delta, (y : A), (x : B) \vdash Q :: (z : C)}{\mathcal{V}\ ;\ \Delta, (x : A \otimes B) \vdash (y \leftarrow \mathsf{recv}\ x\ ;\ Q) :: (z : C)}\ \otimes L$$

Operationally, the provider $(\mathsf{send}\ c\ d\ ;\ P)$ sends the channel $d$ and the continuation channel $c'$ along $c$ as a message and continues with executing $P$. The client receives channel $d$ and continuation channel $c'$ appropriately substituting them.

$$(\otimes S) : \mathsf{proc}(c, \mathsf{send}\ c\ d\ ;\ P) \ \mapsto \mathsf{proc}(c', P[c'/c]), \mathsf{msg}(c, \mathsf{send}\ c\ d\ ;\ c \leftrightarrow c')$$
$$(\otimes C) : \mathsf{msg}(c, \mathsf{send}\ c\ d\ ;\ c \leftrightarrow c'), \mathsf{proc}(e, x \leftarrow \mathsf{recv}\ c\ ;\ Q) \ \mapsto\ \mathsf{proc}(e, Q[c', d/c, x])$$

The dual operator $A \multimap B$ allows the provider to receive a channel of type $A$ and continue to provide type $B$. The client of $A \multimap B$, on the other hand, sends the channel of type $A$ and continues to use $B$ using dual process terms as $\otimes$.

The type $\mathbf{1}$ indicates *termination* requiring that the provider of $x : \mathbf{1}$ send a *close* message, formally written as $\mathsf{close}\ x$ followed by terminating the communication. Correspondingly, the client of $x : \mathbf{1}$ uses the term $\mathsf{wait}\ x\ ;\ Q$ to wait for $x$ to terminate before continuing with executing $Q$.

A forwarding process $x \leftrightarrow y$ identifies the channels $x$ and $y$ so that any further communication along either $x$ or $y$ will be along the unified channel. Its typing rule corresponds to the logical rule of identity.

$$\frac{}{\mathcal{V}\ ;\ y : A \vdash (x \leftrightarrow y) :: (x : A)}\ \mathsf{id}$$

Operationally, a process $c \leftrightarrow d$ *forwards* any message M that arrives on $d$ to $c$ and vice-versa. Since channels are used linearly, the forwarding process can then terminate, ensuring proper renaming, as exemplified in the rules below.

$$(\mathsf{id}^+C) : \mathsf{msg}(d', M), \mathsf{proc}(c, c \leftrightarrow d) \;\mapsto\; \mathsf{msg}(c, M[c/d])$$
$$(\mathsf{id}^-C) : \mathsf{proc}(c, c \leftrightarrow d), \mathsf{msg}(e, M(c)) \;\mapsto\; \mathsf{msg}(e, M(c)[d/c])$$

We write $M(c)$ to indicate that $c$ must occur in message $M$ ensuring that $M$ is the sole client of $c$.

***Process Definitions*** Process definitions have the form $\Delta \vdash f[\overline{\alpha}] = P :: (x : A)$ where $f$ is the name of the process and $P$ its definition, with $\Delta$ being the channels used by $f$ and $x : A$ being the offered channel. In addition, $\overline{\alpha}$ is a sequence of type variables that $\Delta$, $P$ and $A$ can refer to. These type variables are implicitly universally quantified at the outermost level and represent prenex polymorphism. All definitions are collected in the fixed global signature $\Sigma$. For a *valid signature*, we require that $\overline{\alpha}\ ;\ \Delta \vdash P :: (x : A)$ for every definition, thereby allowing definitions to be mutually recursive. A new instance of a defined process $f$ can be spawned with the expression $x \leftarrow f[\overline{A}]\ \overline{y}\ ;\ Q$ where $\overline{y}$ is a sequence of channels matching the antecedents $\Delta$ and $\overline{A}$ is a sequence of types matching the type variables $\overline{\alpha}$. The newly spawned process will use all variables in $\overline{y}$ and provide $x$ to the continuation $Q$.

$$\frac{\begin{array}{c}\overline{y' : B'} \vdash f[\overline{\alpha}] = P_f :: (x' : B) \in \Sigma \\ \Delta' = \overline{(y : B')}[\overline{A}/\overline{\alpha}] \quad \mathcal{V}\ ;\ \Delta, (x : B[\overline{A}/\overline{\alpha}]) \vdash Q :: (z : C)\end{array}}{\mathcal{V}\ ;\ \Delta, \Delta' \vdash (x \leftarrow f[\overline{A}]\ \overline{y}\ ;\ Q) :: (z : C)}\ \mathsf{def}$$

The declaration of $f$ is looked up in the signature $\Sigma$ (first premise), and $\overline{A}$ is substituted for $\overline{\alpha}$ while matching the types in $\Delta'$ and $\overline{y}$ (second premise). Similarly, the freshly created channel $x$ has type $A$ from the signature with $\overline{A}$ substituted for $\overline{\alpha}$.

The complete set of rules for the type system and the operational semantics for our language are presented in [14].

#### 6.2 Type Safety

The extension of session types with nested polymorphism is proved type safe by the standard theorems of *preservation* and *progress*, also known as *session fidelity* and *deadlock freedom*. At runtime, a program is represented using a multiset of semantic objects denoting processes and messages defined as a *configuration*.

$$\mathcal{S} \;::=\; \cdot \mid \mathcal{S}, \mathcal{S}' \mid \mathsf{proc}(c, P) \mid \mathsf{msg}(c, M)$$

We say that $\mathsf{proc}(c, P)$ (or $\mathsf{msg}(c, M)$) provide channel $c$. We stipulate that no two distinct semantic objects in a configuration provide the same channel.

***Type Preservation*** The key to preservation is defining the rules to *type a configuration*. We define a well-typed configuration using the judgment $\Delta_1 \vDash_{\Sigma} \mathcal{S} :: \Delta_2$ denoting that configuration $\mathcal{S}$ uses channels $\Delta_1$ and provides channels $\Delta_2$. A configuration is always typed w.r.t. a valid signature $\Sigma$. Since the signature $\Sigma$ is fixed, we elide it from the presentation.

$$\frac{}{\Delta \vDash (\cdot) :: \Delta}\ \mathsf{emp} \qquad \frac{\Delta_1 \vDash \mathcal{S}_1 :: \Delta_2 \qquad \Delta_2 \vDash \mathcal{S}_2 :: \Delta_3}{\Delta_1 \vDash (\mathcal{S}_1, \mathcal{S}_2) :: \Delta_3}\ \mathsf{comp}$$

$$\frac{\cdot\, ;\, \Delta \vdash P :: (x : A)}{\Delta \vDash \mathsf{proc}(x, P) :: (x : A)}\ \mathsf{proc} \qquad \frac{\cdot\, ;\, \Delta \vdash M :: (x : A)}{\Delta \vDash \mathsf{msg}(x, M) :: (x : A)}\ \mathsf{msg}$$

**Fig. 2.** Typing rules for a configuration

The rules for typing a configuration are defined in Figure 2. The $\mathsf{emp}$ rule states that an empty configuration does not consume any channels provides all channels it uses. The $\mathsf{comp}$ rule composes two configurations $\mathcal{S}_1$ and $\mathcal{S}_2$; $\mathcal{S}_1$ provides channels $\Delta_2$ while $\mathcal{S}_2$ uses channels $\Delta_2$. The rule $\mathsf{proc}$ creates a singleton configuration out of a process. Since configurations are runtime objects, they do not refer to any free variables and $\mathcal{V}$ is empty. The $\mathsf{msg}$ rule is analogous.

***Global Progress*** To state progress, we need to define a *poised process* [39]. A process $\mathsf{proc}(c, P)$ is poised if it is trying to receive a message on $c$. Dually, a message $\mathsf{msg}(c, M)$ is poised if it is sending along $c$. A configuration is poised if every message or process in the configuration is poised. Intuitively, this represents that the configuration is trying to communicate *externally* along one of the channels it uses or provides.

**Theorem 6 (Type Safety).** *For a well-typed configuration* $\Delta_1 \vDash_\Sigma \mathcal{S} :: \Delta_2$,

*(i) (Preservation) If* $\mathcal{S} \mapsto \mathcal{S}'$, *then* $\Delta_1 \vDash_\Sigma \mathcal{S}' :: \Delta_2$
*(ii) (Progress) Either* $\mathcal{S}$ *is poised, or* $\mathcal{S} \mapsto \mathcal{S}'$.

*Proof.* Preservation is proved by case analysis on the rules of operational semantics. First, we invert the derivation of the current configuration $\mathcal{S}$ and use the premises to assemble a new derivation for $\mathcal{S}'$. Progress is proved by induction on the right-to-left typing of $\mathcal{S}$ so that either $\mathcal{S}$ is empty (and therefore poised) or $\mathcal{S} = (\mathcal{D}, \mathsf{proc}(c, P))$ or $\mathcal{S} = (\mathcal{D}, \mathsf{msg}(c, M))$. By the induction hypothesis, either $\mathcal{D} \mapsto \mathcal{D}'$ or $\mathcal{D}$ is poised. In the former case, $\mathcal{S}$ takes a step (since $\mathcal{D}$ does). In the latter case, we analyze the cases for $P$ and $M$, applying multiple steps of inversion to show that in each case either $\mathcal{S}$ can take a step or is poised.

### 7 Relationship to Context-Free Session Types

As ordinarily formulated, session types express communication protocols that can be described by regular languages [45]. In particular, the type structure is necessarily tail recursive. Context-free session types (CFSTs) were introduced by Thiemann and Vascoconcelos [45] as a way to express a class of communication protocols that are not limited to tail recursion. CFSTs express protocols that can be described by single-state, real-time DPDAs that use the empty stack acceptance criterion [1,34].

Despite their name, the essence of CFSTs is not their connection to a particular subset of the (deterministic) context-free languages. Rather, the essence of CFSTs is that session types are enriched to admit a notion of sequential composition. Nested session types are strictly more expressive than CFSTs, in the sense that there exists a proper fragment of nested session types that is closed under a notion of sequential composition. (In keeping with process algebras like ACP [2], we define a sequential composition to be an operation that satisfies the laws of a right-distributive monoid.)

Consider (up to $\alpha$,$\beta$,$\eta$-equivalence) the linear, tail functions from types to types with unary type constructors only:

$$\begin{aligned} S, T ::= {} & \hat{\lambda}\alpha.\, \alpha \mid \hat{\lambda}\alpha.\, V[S\,\alpha] \mid \hat{\lambda}\alpha.\, \oplus\{\ell : S_\ell\,\alpha\}_{\ell \in L} \mid \hat{\lambda}\alpha.\, \&\{\ell : S_\ell\,\alpha\}_{\ell \in L} \\ & \mid \hat{\lambda}\alpha.\, A \otimes (S\,\alpha) \mid \hat{\lambda}\alpha.\, A \multimap (S\,\alpha) \end{aligned}$$

The linear, tail nature of these functions allows the type $\alpha$ to be thought of as a continuation type for the session. The functions $S$ are closed under function composition, and the identity function, $\hat{\lambda}\alpha.\, \alpha$, is included in this class of functions. Moreover, because these functions are tail functions, composition right-distributes over the various logical connectives in the following sense:

$$\begin{aligned} (\hat{\lambda}\alpha.\, V[S\,\alpha]) \circ T &= \hat{\lambda}\alpha.\, V[(S \circ T)\,\alpha] \\ (\hat{\lambda}\alpha.\, \oplus\{\ell : S_\ell\,\alpha\}_{\ell \in L}) \circ T &= \hat{\lambda}\alpha.\, \oplus\{\ell : (S_\ell \circ T)\,\alpha\}_{\ell \in L} \\ (\hat{\lambda}\alpha.\, A \otimes (S\,\alpha)) \circ T &= \hat{\lambda}\alpha.\, A \otimes ((S \circ T)\,\alpha) \end{aligned}$$

and similarly for $\&$ and $\multimap$. Together with the monoid laws of function composition, these distributive properties justify defining sequential composition as $S;T = S \circ T$.

This suggests that although many details distinguish our work from CFSTs, nested session types cover the essence of sequential composition underlying context-free session types. However, even stating a theorem that every CFST process can be translated into a well-typed process in our system of nested session types is difficult because the two type systems differ in many details: we include $\otimes$ and $\multimap$ as session types, but CFSTs do not; CFSTs use a complex kinding system to incorporate unrestricted session types and combine session types with ordinary function types; the CFST system uses classical typing for session types and a procedure of type normalization, whereas our types are intuitionistic and do not rely on normalization; and the CFST typing rules are based on natural deduction, rather than the sequent calculus. With all of these differences, a formal translation, theorem, and proof would not be very illuminating beyond the essence already described here. Empirically, we can also give analogues of the published examples for CFSTs (see, e.g., the first two examples of Section 9).

Finally, nested session types are strictly *more* expressive than CFSTs. Recall from Section 2 the language $L_3 = \{\mathbf{L}^n \mathbf{a}\, \mathbf{R}^n \mathbf{a} \cup \mathbf{L}^n \mathbf{b}\, \mathbf{R}^n \mathbf{b} \mid n > 0\}$, which can be expressed using nested session types with *two* type parameters used in an essential way. Moreover, Korenjak and Hopcroft [34] observe that this language cannot be recognized by a single-state, real-time DPDA that uses empty stack acceptance, and thus, CFSTs cannot express the language $L_3$. More broadly, nested types allow for finitely many states and acceptance by empty stack or final state, while CFSTs only allow a single state and empty stack acceptance.

## 8 Implementation

We have implemented a prototype for nested session types and integrated it with the open-source Rast system [17]. Rast (Resource-aware session types) is a programming language which implements the intuitionistic version of session types [7] with support for arithmetic refinements [18], ergometric [16] and temporal [15] types for complexity analysis. Our prototype extension is implemented in Standard ML (8011 lines of code) containing a lexer and parser (1214 lines), a type checker (3001 lines) and an interpreter (201 lines) and is well-documented. The prototype is available in the Rast repository [13].

***Syntax*** A program contains a series of mutually recursive type and process declarations and definitions, concretely written as

```
type V[x1]...[xk] = A
decl f[x1]...[xk] : (c1 : A1) ... (cn : An) |- (c : A)
proc c <- f[x] c1 ... cn = P
```

Type $V[\overline{x}]$ is represented in concrete syntax as `V[x1]...[xk]`. The first line is a *type definition*, where $V$ is the type name parameterized by type variables $x_1, \ldots, x_k$ and $A$ is its definition. The second line is a *process declaration*, where $f$ is the process name (parameterized by type variables $x_1, \ldots, x_k$), $(c_1 : A_1) \ldots (c_n : A_n)$ are the used channels and corresponding types, while the offered channel is $c$ of type $A$. Finally, the last line is a *process definition* for the same process $f$ defined using the process expression $P$. We use a hand-written lexer and shift-reduce parser to read an input file and generate the corresponding abstract syntax tree of the program. The reason to use a hand-written parser instead of a parser generator is to anticipate the most common syntax errors that programmers make and respond with the best possible error messages.

Once the program is parsed and its abstract syntax tree is extracted, we perform a *validity check* on it. This includes checking that type definitions, and process declarations and definitions are closed w.r.t. the type variables in scope. To simplify and improve the efficiency of the type equality algorithm, we also assign internal names to type subexpressions parameterized over their free index variables. These internal names are not visible to the programmer.

***Type Checking and Error Messages*** The implementation is carefully designed to produce precise error messages. To that end, we store the extent (source location) information with the abstract syntax tree, and use it to highlight the source of the error. We also follow a bi-directional type checking [40] algorithm reconstructing intermediate types starting with the initial types provided in the declaration. This helps us precisely identify the source of the error. Another particularly helpful technique has been *type compression.* Whenever the type checker expands a type $V[\overline{A}]$ defined as $V[\overline{\alpha}] = B$ to $B[\overline{A}/\overline{\alpha}]$ we record a reverse mapping from $B[\overline{A}/\overline{\alpha}]$ to $V[\overline{\alpha}]$. When printing types for error messages this mapping is consulted, and complex types may be compressed to much simpler forms, greatly aiding readability of error messages.

### 9 More Examples

***Expression Server*** We adapt the example of an arithmetic expression from prior work on context-free session types [45]. The type of the server is defined as

```
type bin = +{ b0 : bin, b1 : bin, $ : 1 }
type tm[K] = +{ const : bin * K,
                add : tm[tm[K]],
                double : tm[K] }
```

The type `bin` represents a constant binary natural number. A process *providing* a binary number sends a stream of bits, `b0` and `b1`, starting with the least significant bit and eventually terminated by `$`.

An arithmetic term, parameterized by continuation type `K` can have one of three forms: a constant, the sum of two terms, or the double of a term. Consequently, the type `tm[K]` ensures that a process providing `tm[K]` is a *well-formed term*: it either sends the `const` label followed by sending a constant binary number of type `bin` and continues with type `K`; or it sends the `add` label and continues with `tm[tm[K]]`, where the two terms denote the two summands; or it sends the `double` label and continues with `tm[K]`. In particular, the continuation type `tm[tm[K]]` in the `add` branch enforces that the process must send exactly two summands for sums.

As a first illustration, consider two binary constants $a$ and $b$, and suppose that we want to create the expression $a + 2b$. We can issue commands to the expression server in a *prefix notation* to obtain $a + 2b$, as shown in the following `exp[K]` process, which is parameterized by a continuation type `K`.

```
decl exp[K] : (a : bin) (b : bin) (k : K) |- (e : tm[K])
proc e <- exp[K] a b k =
  e.add ; e.const ; send e a ; % (b:bin) (k:K) |- (e : tm[K])
  e.double ; e.const ; send e b ; % (k:K) |- (e : K)
  e <-> k
```

In prefix notation, $a + 2b$ would be written $+\ (a)\ (2\ b)$, which is exactly the form followed by the `exp` process: The process sends `add`, followed by `const` and the number `a`, followed by `double`, `const`, and `b`. Finally, the process continues at type `K` by forwarding `k` to `e` (intermediate typing contexts on the right).

To evaluate a term, we can define an `eval` process, parameterized by type `K`:

```
decl eval[K] : (t : tm[K]) |- (v : bin * K)
```

The `eval` process uses channel `t : tm[K]` as argument, and offers `v : bin * K`. The process evaluates term `t` and sends its binary value along `v`. The technical report contains the full implementation [14].

***Serializing binary trees*** Another example from [45] is serializing binary trees. Here we adapt that example to our system. Binary trees can be described by:

```
type Tree[a] = +{ node : Tree[a] * a * Tree[a] , leaf : 1 }
```

These trees are polymorphic in the type of data stored at each internal node. A tree is either an internal node or a leaf, with the internal nodes storing channels that emit the left subtree, data, and right subtree. Owing to the multiple channels stored at each node, these trees do not exist in a serial form.

We can, however, use a different type to represent serialized trees:

```
type STree[a][K] = +{ nd : STree[a][a * STree[K]] , lf : K }
```

A serialized tree is a stream of node and leaf labels, `nd` and `lf`, parameterized by a continuation type `K`. Like `add` in the expression server, the label `nd` continues with type `STree[a][a * STree[K]]`: the label `nd` is followed by the serialized left subtree, which itself continues by sending the data stored at the internal node and then the serialized right subtree, which continues with type `K`.[3]

Using these types, it is relatively straightforward to implement processes that serialize and deserialize such trees. The process `serialize` can be declared with:

```
decl serialize[a][K] : (t : Tree[a]) (k : K) |- (s : STree[a][K])
```

This process uses channels `t` and `k` that hold the tree and continuation, and offers that tree's serialization along channel `s`. If the tree is only a leaf, then the process forwards to the continuation. Otherwise, if the tree begins with a node, then the serialization begins with `nd`. A recursive call to `serialize` serves to serialize the right subtree with the given continuation. A subsequent recursive call serializes the left subtree with the data together with the right subtree's serialization as the new continuation.

It is also possible to implement a process for deserializing trees, but because of space limitations, we will not describe `deserialize` here.

```
decl deserialize[a][K] : (s : STree[a][K]) |- (tk : Tree[a] * K)
```

***Generalized tries for binary trees*** Using nested types in Haskell, prior work [28] describes an implementation of generalized tries that represent mappings on binary trees. Our type system is expressive enough to represent such generalized tries. We can reuse the type `Tree[a]` of binary trees given above. The type `Trie[a][b]` describes tries that represent mappings from `Tree[a]` to type `b`:

[3] The presence of `a *` means that this is not a true serialization because it sends a separate channel along which the data of type `a` is emitted. But there is no uniform mechanism for serializing polymorphic data, so this is as close to a true serialization as possible. Concrete instances of type `Tree` with, say, data of base type `int` could be given a true serialization by "inlining" the data of type `int` in the serialization.

```
type Trie[a][b] = &{ lookup_leaf : b ,
                     lookup_node : Trie[a][a -o Trie[a][b]] }
```

A process for looking up a tree in such tries can be declared by:

```
decl lookup_tree[a][b] : (m : Trie[a][b]) (t : Tree[a]) |- (v : b)
```

To lookup a tree in a trie, first determine whether that tree is a `leaf` or a `node`. If the tree is a `leaf`, then sending `lookup_leaf` to the trie will return the value of type `b` associated with that tree in the trie.

Otherwise, if the tree is a `node`, then sending `lookup_node` to the trie results in a trie of type `Trie[a][a -o Trie[a][b]]` that represents a mapping from left subtrees to type `a -o Trie[a][b]`. We then lookup the left subtree in this trie, resulting in a process of type `a -o Trie[a][b]` to which we send the data stored at our original tree's root. That results in a trie of type `Trie[a][b]` that represents a mapping from right subtrees to type `b`. Therefore, we finally lookup the right subtree in this new trie and obtain a result of type `b`, as desired.

We can define a process that constructs a trie from a function on trees:

```
decl build_trie[a][b] : (f : Tree[a] -o b) |- (m : Trie[a][b])
```

Both `lookup_tree` and `build_trie` can be seen as analogues to `deserialize` and `serialize`, respectively, converting a lower-level representation to a higher-level representation and vice versa. These types and declarations mean that tries represent total mappings; partial mappings are also possible, at the expense of some additional complexity.

All our examples have been implemented and type checked in the open-source Rast repository [13]. We have also further implemented the standard polymorphic data structures such as lists, stacks and queues.

### 10 Further Related Work

To our knowledge, our work is the first proposal of polymorphic recursion using nested type definitions in session types. Thiemann and Vasconcelos [45] use polymorphic recursion to update the channel between successive recursive calls but do not allow type constructors or nested types. An algorithm to check type equivalence for the non-polymorphic fragment of context-free session types has been proposed by Almeida et al. [1].

Other forms of polymorphic session types have also been considered in the literature. Gay [24] studies bounded polymorphism associated with branch and choice types in the presence of subtyping. He mentions recursive types (which are used in some examples) as future work, but does not mention parametric type definitions or nested types. Bono and Padovani [4,5] propose (bounded) polymorphism to type the endpoints in copyless message-passing programs inspired by session types, but they do not have nested types. Following Kobayashi's approach [33], Dardha et al. [12] provide an encoding of session types relying on linear and variant types and present an extension to enable parametric and bounded polymorphism (to which recursive types were added separately [11]) but not parametric type definitions nor nested types. Caires et al. [6] and Perez et al. [38] provide behavioral polymorphism and a relational parametricity principle for session types, but without recursive types or type constructors.

Nested session types bear important similarities with first-order cyclic terms, as observed by Jančar. Jančar [30] proves that the trace equivalence problem of first-order grammars is decidable, following the original ideas by Stirling for the language equality problem in deterministic pushdown automata [43]. These ideas were also reformulated by Sénizergues [41]. Henry and Sénizergues [27] proposed the only practical algorithm to decide the language equivalence problem on deterministic pushdown automata that we are aware of. Preliminary experiments show that such a generic implementation, even if complete in theory, is a poor match for the demands made by our type checker.

### 11 Conclusion

Nested session types extend binary session types with parameterized type definitions. This extension enables us to express polymorphic data structures just as naturally as in functional languages. The proposed types are able to capture sequences of communication actions described by deterministic context-free languages recognized by deterministic pushdown automata with several states, that accept by empty stack or by final state. In this setting, we show that type equality is decidable. To offset the complexity of type equality, we give a practical type equality algorithm that is sound, efficient, but incomplete.

In the future, we are planning to explore subtyping for nested types. In particular, since the language inclusion problem for simple languages [22] is undecidable, we believe subtyping can be reduced to inclusion and would also be undecidable. Despite this negative result, it would be interesting to design an algorithm to approximate subtyping. That would significantly increase the programs that can be type checked in the system. In another direction, since Rast [17] supports arithmetic refinements for lightweight verification, it would be interesting to explore how refinements interact with polymorphic type parameters, namely in the presence of subtyping. We would also like to explore examples where the current type equality is not adequate. Finally, protocols in distributed algorithms such as consensus or leader election (Raft, Paxos, etc.) depend on unbounded memory and cannot usually be expressed with finite control structure. In future work, we would like to see if these protocols can be expressed with nested session types.

*Acknowledgements.* Support for this research was provided by the Fundação para a Ciência e a Tecnologia (Portuguese Foundation for Science and Technology) through the Carnegie Mellon Portugal Program – Visiting Faculty Program and through the LASIGE Research Unit, ref. UIDB/00408/2020, and by the National Science Foundation under SaTC Award 1801369, CAREER Award 1845514 and Grant No. 1718276.
